# Psychedelic Therapy, Positive Emotional Experiences, and the Central Role of Self-Compassion

**DOI:** 10.21203/rs.3.rs-7420529/v1

**Published:** 2025-08-22

**Authors:** Richard Zeifman, George Danias, Gabrielle Agin-Liebes, Broc Pagni, Hannes Kettner, Venkat Bhat, Stephen Ross, David Erritzoe, Robin Carhart-Harris

**Affiliations:** The New School for Social Research; NYU Grossman School of Medicine; Yale School of Medicine; NYU Grossman School of Medicine; University of California San Francisco; Unity Health Toronto; NYU Grossman School of Medicine; Imperial College London; UCSF

**Keywords:** psychedelics, psilocybin, positive emotions, self-compassion, positive mood, mystical experience, mental health

## Abstract

**Background:**

Psychedelics can acutely induce mystical experiences and elevated positive mood, which may contribute to the potential benefits of psychedelic therapy. However, there remains limited understanding of the occurrence and importance of specific positive emotional experiences within psychedelic therapy. Therefore, we examined the effects of psychedelics on positive emotional experiences and their association with improvements in mental health.

**Methods:**

Study 1 was an observational study of naturalistic psychedelic use. Study 2 used data from a clinical trial that compared psilocybin with escitalopram in individuals with major depressive disorder. In this trial, participants completed two dosing sessions, where they received either 25mg or 1mg of psilocybin. In both studies, following their psychedelic experience or psilocybin dosing sessions, participants rated their acute experiences of seven specific positive emotional experiences (self-compassion, compassion toward others, gratitude, love, awe, ecstasy, and peace).

**Results:**

Relative to low-dose psychedelic, medium and high-dose psychedelic use were associated with greater positive emotional experiences. Relative to 1mg psilocybin, 25mg psilocybin was associated with greater positive emotional experiences. Several positive emotional experiences predicted improvements in mental health and mediated treatment outcomes, with the strongest evidence for the effect of self-compassion (over and above mystical experience and positive mood).

**Discussion:**

Positive emotional experiences, especially self-compassion, appear to play an important role within psychedelic therapy. Based on these findings, we highlight key considerations surrounding psychotherapeutic approaches to, and optimization of, psychedelic therapy. Future research should move beyond retrospective, self-reports of emotional experiences to fully capture their role within psychedelic therapy.

## Introduction

Classic psychedelics have a wide range of acute effects on conscious experience, including alterations in perception (e.g., visual distortions), sense of self (e.g., ego dissolution), and affect (e.g., positive mood). However, there remains limited understanding of the acute effects of psychedelics on specific positive emotions (e.g., self-compassion, compassion toward others, awe, love, ecstasy, gratitude, and peace). Moreover, while psychedelic therapy shows potential mental health benefits (Yao et al., 2024), there is an ongoing debate over whether acute psychedelic experiences contribute to these benefits (Kush et al., 2025). Although research has generally focused on the role of acute mystical experiences and positive mood in mediating psychedelic therapy outcomes (Romeo et al., 2025), there remains limited understanding of the importance of specific positive emotions within psychedelic therapy (Goldy et al., 2024). Accordingly, the present manuscript examines the effects of psychedelics on (a) the experience of specific positive emotions; and (b) their importance in facilitating psychedelic-related mental health benefits.

### Psychedelics and Acute Positive Emotions

Psychedelics can acutely increase positive mood among healthy (e.g., Griffiths et al., 2006, 2011, 2018, 2025) and clinical (e.g., Holze et al., 2023; Griffiths et al., 2016) samples. However, there is limited research on the effects of psychedelics on specific positive emotions (Goldy et al., 2024), which is important because there are key differences between the distinct subjective experience of specific emotions (Cowen & Keltner, 2018; Weidman & Tracy, 2020); ways in which they can be targeted within treatment; and their long-term effects on mental health (Greenberg, 2004). Using observer-rated measures, several studies have found that psilocybin elicits feelings of peace/harmony and joy/intense happiness among healthy individuals (Carbonaro et al., 2018; Griffiths et al., 2006, 2011, 2018). Additionally, a lab-based study found that microdoses of psilocybin acutely increases awe (van Elk et al., 2022). Qualitative research has also identified psychedelic-induced experiences of specific positive emotions, such as self-compassion, compassion toward others, awe, gratitude, and love, within clinical (Agin-Liebes, Ekman et al., 2024; Agin-Liebes, Nielson et al., 2024; Bogenschutz et al., 2018; Malone et al., 2018; Noorani et al., 2018; Renelli et al., 2020) and non-clinical samples (Brouwer et al., 2025; Michael et al., 2023; Tsupari et al., 2025). However, research has not yet quantitatively examined the effect of psychedelics on self-reported experiences of specific positive emotions following a medium-to-high dose administration of a psychedelic (i.e., doses with existing evidence for their therapeutic benefit) or within a clinical sample.

### Acute Psychedelic Experiences and Mental Health Outcomes

There is an ongoing debate surrounding whether acute subjective psychedelic effects have any relevance to the potential therapeutic benefits of psychedelics (Kush et al., 2025). To date, the acute experience that has received the most attention within psychedelic research is the mystical (or unitive) experience. The intensity of the mystical experience often (though not always; e.g., Calder et al., 2024; Zeifman, Wagner et al., 2023) predicts or mediates improvements in mental health (e.g., Goodwin et al., 2025; Murphy et al., 2022; Ross et al., 2016; Timmerman et al., 2024). However, the mystical experience construct is challenging to operationalize and includes multiple non-discrete elements. Focusing narrowly on the mystical (or other non-ordinary) experience within psychedelic therapy may also increase blind breaking; confuse patients and treatment providers who are unfamiliar with such experiences; and interfere with treatment benefits among the many individuals that are less open to spiritual/religious experiences (Nicholas et al., 2018) or do not have a “complete” mystical experience (Liechti et al., 2017). Exclusive focus on mystical experiences is also a form of psychedelic exceptionalism and may fail to identify overlapping mechanisms between psychedelic therapy and other therapeutic interventions (Aicher et al., 2024). Accordingly, there is a need for exploring the importance of additional elements of the acute psychedelic experience. In light of qualitative findings from psychedelic therapy trials (e.g., Agin-Liebes, Nielson et al., 2024) and the growing support for the importance of positive emotional experiences within mental health interventions (Craske et al., 2024; Garland et al., 2010), further understanding the role of specific positive emotions within psychedelic therapy may help to tailor and optimize treatment delivery.

#### Positive Emotions and Post-Psychedelic Mental Health Outcomes

A subcomponent of the mystical experience is positive mood. Some psychedelic therapy research has found significant associations between acute experiences of positive mood and therapeutic outcomes (Irrmischer et al., 2025; McCulloch et al., 2022; Weiss et al., 2024), while others have not (Palhano-Fontes et al., 2019; von Rotz et al., 2023). Importantly, previously used measures of positive mood collapse across positive emotional experiences and do not include items that assess specific positive emotional experiences, such as self-compassion, compassion or gratitude. Researchers have suggested that specific acute positive emotional experiences (Goldy et al., 2024; Newton & Moreton, 2023), including awe (Hendricks, 2018) and self-compassion (Johansen et al., 2023), may play an important role in mediating psychedelic therapy outcomes. In line with these suggestions, qualitative research from clinical trials has highlighted the therapeutic importance of several positive emotions (e.g., self-compassion, compassion toward others awe, gratitude, and love) within psychedelic therapy (Agin-Liebes, Ekman et al., 2024; Agin-Liebes, Nielson et al., 2024; Bogenschutz et al., 2018; Malone et al., 2018; Noorani et al., 2018; Renelli et al., 2020). A recent naturalistic study of psilocybin therapy found that relaxation (but not mystical experience) predicted reductions in depressive symptoms (Calder et al., 2024). Additionally, among individuals with treatment-resistant depression who received psilocybin therapy, Roseman et al. (2018) found associations between self-reported experiences of peace, joy, and awe (but not love) and reductions in depressive symptoms. Similarly, a prospective observational study found that experience of awe following ayahuasca administration predicted decreases in state and trait anxiety (but not depression severity; Lowe et al., 2024). Nonetheless, there remains limited quantitative research on the association between specific positive emotional experiences (especially self-compassion) and psychedelic therapy-related mental health outcomes; nor the importance of these specific positive emotions relative to mystical experience and positive mood.

In summary, there remains limited understanding surrounding the specific positive emotions that are enhanced by psychedelic administration and their importance within psychedelic therapy. Therefore, the present research examines: (a) the effect of psychedelics on acute experiences of specific positive emotions (self-compassion, compassion, gratitude, love, awe, ecstasy, and peace); (b) the relationship between these positive emotions and mental health outcomes following psychedelic use/psilocybin therapy; and (c) the importance of these emotions in predicting mental health outcomes relative to mystical experience and positive mood. We first examined these questions in the context of a naturalistic observational study of psychedelic use (Study 1) and then sought to replicate and extend our findings using data from a clinical trial of psilocybin therapy (Study 2).

## Study 1 (Prospective Observational Study): Methods

### Procedures and Participants

This study was a prospective cohort study using an online convenience sample of individuals planning to use a psychedelic. Participants were recruited via online advertisements shared through social media (e.g., Facebook, Twitter), email newsletters, and online forums (e.g., Reddit). A software platform (https://www.psychedelicsurvey.com/) was used to inform prospective subjects about the trial and informed consent, enroll them in an emailing system to remind them of study timepoints, and contained links to relevant surveys, which were implemented and hosted by the online service system Survey Gizmo (now alchemer). The study received approval from Imperial College London’s Imperial College Research Ethics Committee (ICREC) and the Joint Research Compliance Office (JRCO). Further details about the study procedure can be found in Haijen et. al. (2018).

For the original study (Haijen et al., 2018), the inclusion criteria were: (a) being ≥18 years old; (b) comprehension of English; and (c) an intention to use a psychedelic (i.e., psilocybin/ magic mushrooms/truffles, LSD/1P-LSD, ayahuasca, DMT/5-MeODMT, salvia divinorum, mescaline, or iboga/ibogaine). For the present analyses, participants were excluded if they failed to complete all of the measures related to their acute psychedelic experience or reported using a drug other than a serotonergic psychedelic (e.g., iboga, salvia, ketamine, cannabis, or 3,4-Methylenedioxymethamphetamine [MDMA]). The final sample included 359 individuals. Based on when individuals reported planning to use a psychedelic in the future, they were sent online surveys at three key time points: 1 week before psychedelic use (baseline); the day after their psychedelic session, to assess details around their psychedelic use and acute effects; and 4 weeks after psychedelic use, to assess changes in well-being.

### Measures

#### Demographics

Participants provided demographic information such as age, sex, nationality, native language, educational background, and employment status.

#### Psychedelic Use

The day after their psychedelic use, participants were asked to identify which psychedelic they used and to provide an estimate of the total amount of the psychedelic that they used (in reference to a typical dose of LSD): low dose (<50μg LSD); moderate dose (<100μg LSD); high dose (<200μg LSD); very high dose (<300μg LSD); or extremely high dose (>300μg LSD). For analytic purposes, doses identified as high, very high, or extremely high were considered high doses.

#### Mystical Experience and Positive Mood

The day after their psychedelic use, participants completed the MEQ-30 (MacLean et al., 2012), a 30-item self-report measure of the intensity of a mystical experience. The MEQ-30 includes a total score and four subscales: (1) *Mystical* (reflecting experiences of unity, noetic quality, and sacredness); (2) *Positive Mood*; (3) *Transcendence of Time and Space*; and (4) *Ineffability*. Participants rated how strongly each item reflected their experience during the dosing session using a scale from 0 (“none/not at all”) to 5 (“extreme; more than any other time in my life”). In line with past research (Roseman et al., 2019; Zeifman, Kettner et al., 2023), items were then multiplied by 20 to allow for calculating mean scores (MEQ total and Positive Mood subscale) ranging from 0–100.

#### Specific Positive Emotional Experiences

Acute positive emotions during participants’ psychedelic experiences were measured the day after psychedelic use. Self-compassion (“I felt compassion towards myself”), compassion toward others (“I felt compassion towards others”), gratitude (“I felt a general sense of gratitude”), love (“I felt a general sense of love”), and awe (“I experienced a general sense of awe”) were measured using items constructed for the present study. Peace was measured using a single item (“I experienced profound inner peace.”) from the Altered States of Consciousness questionnaire (Studerus et al., 2010). Participants were instructed to rate the extent to which these statements applied to their psilocybin experience, compared to their normal waking consciousness. Ecstasy was measured using a single item (“Experience of ecstasy”) from the revised Mystical Experience Questionnaire (MEQ-30; MacLean et al., 2012) on a scale from 0 (none) to 5 (Extreme; more than any other time in my life) and then multiplied by 20 as described above.

#### Well-Being

The Warwick-Edinburgh Mental Wellbeing Scale (WEMWBS) (Tennant et al., 2007) was used to assess psychological well-being. The WEMWBS measures aspects of positive mental health, including positive affect, satisfying interpersonal relationships, and positive functioning. Participants respond to items on a 5-point Likert scale ranging from 1 (None of the time) to 5 (All of the time). The primary timepoints for the WEMWBS were before psychedelic use and 4 weeks after psychedelic use.

### Statistical Analyses

A multivariate analysis of variance (MANOVA) examined the effect of psychedelic dose on acute experiences of specific positive emotions. Post-hoc comparisons assessed differences between low, medium, and high doses. We used the false discovery rate (FDR) correction for multiple comparisons (Benjamini & Hochberg, 1995) for main effects of dose and then for all post-hoc contrasts. Effect sizes are reported using partial eta squared (*ηp*^*2*^). An exploratory MANOVA examined the effect of specific psychedelic compounds (LSD, psilocybin, and ayahuasca only due to limited sample sizes for other compounds) on positive emotional experiences. Dosage was included as a covariate. FDR-corrected post-hoc comparisons assessed the effect of psychedelic compounds for each specific positive emotion, while uncorrected pairwise comparisons assessed differences between the psilocybin, LSD, and ayahuasca groups.

Pearson correlation analyses examined the relationships between acute positive emotional experiences and residualized change in well-being at 4 weeks post-psychedelic use. Next, an exploratory linear regression analysis simultaneously included all of the positive emotions as predictors of residualized change in well-being. In two additional models, mystical experience and positive mood were also added separately to the model to examine the unique importance of specific positive emotions in predicting improvements in well-being. For the Pearson correlation and regression analyses, we generated bias-corrected and accelerated (BCa) 95% confidence intervals (CI) using bootstrapping (with 5,000 resamples).

All statistical analyses were conducted using SPSS (Version 29.0.2.0). Figures were generated using R-studio (with the following libraries: ggplot2, scales, devtools, ggradar, & fmsb) and edited using GIMP photo-editing software.

## Study 1 (Prospective Observational Study): Results

### Descriptives

Participants’ mean age was 30.60 (SD=11.03). The majority of participants were male (*n*=239; 66.6%) and had a university level of education (*n*=189; 52.6%). They were most commonly working full-time (*n*=125; 34.8% and from the United States (*n*=95; 26.5%). Participants reported using LSD/1P-LSD (*n*=183; 51.0%), psilocybin/magic mushrooms/truffles (*n*=109; 30.4), ayahuasca (*n*=42; 11.7%), DMT/5-MeO-DMT (*n*=12; 3.3%), mescaline (peyote/san pedro; *n*=10; 2.8%), or a combination of these substances (*n*=3; 0.8%). *n*=125; 34.8% for their psychedelic experience. They also reported using the following doses: low dose (*n*=36; 10%); moderate dose (*n*=136; 37.9%); and high dose (*n*=187; 52.1%).

### Experiences of Specific Positive Emotions (Low, Medium, and High Dose Psychedelic Use)

**Figure 1:**
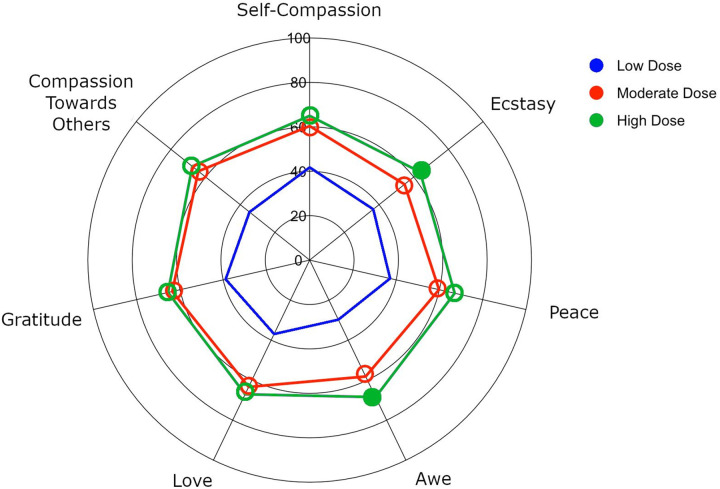
Spider plot demonstrating ratings for each positive emotion at low (blue), moderate (red), and high (green) doses (Study 1). Ratings in the high dose group that differ significantly (*p*<.05) from the low and moderate dose group are marked with filled in circles, while ratings that differ significantly only from the low dose group are marked with empty circles.

There was a significant effect of dose across all positive emotional experiences, Pillai’s Trace=0.16, *F*(14,702)=7.28, *p*<.001, *ηp*^*2*^=.081. Relative to low doses of a psychedelic, medium and high doses were associated with higher levels of self-compassion, compassion toward others, gratitude, love, ecstasy, awe, and peace (with medium-large effect sizes). Relative to medium doses of a psychedelic, high doses were associated with significantly higher levels of awe and ecstasy (with small effect sizes). See Table 1 and Figure 1.

After controlling for dosage (*p*<.001), a significant effect of psychedelic compound on positive emotional experiences was detected (*F*(14,650)=3.10, *p*<.001, *ηp*^*2*^=.063). Positive emotions that survived FDR-correction were love, gratitude, and compassion for others, while self-compassion did not. Pairwise analysis between psychedelic compounds revealed greater reports of gratitude and love after psilocybin and ayahuasca compared to LSD, and greater compassion for others and self-compassion after psilocybin compared to LSD. See Supplementary Table S1.

### Associations Between Positive Emotional Experiences and Post-Psychedelic Well-Being

See Table 2 for correlation and regression analyses. Self-compassion, compassion toward others, gratitude, love, and awe were positively correlated with residualized change in well-being at 4 weeks post-psychedelic use. Ecstasy, peace, mystical experience, and positive mood were not significantly associated with residualized change in well-being. Effect sizes were medium for self-compassion and compassion toward others and small for all other predictors.

In a model that included all of the positive emotional experiences (*F*[7, 138]=3.74, *p*<.001, *R*^*2*^=.16), self-compassion was the only significant predictor of changes in well-being. Adding either mystical experience (*R*^*2*^=.16 *F*_change_=0.48, *p*=.490) or positive mood (*R*^*2*^=.16 *F*_change_=0.49, *p*=.484) to this model did not significantly increase the variance explained and self-compassion remained the only significant predictor.

## Study 2 (Randomized Controlled Trial): Methods

### Trial Design

This was a randomized, double-blind, placebo-controlled trial (Carhart-Harris et al., 2021). The psilocybin group received two 25mg doses of psilocybin alongside daily placebo capsules. The escitalopram group received daily escitalopram (10–20mg) and two 1mg doses of psilocybin (an amount that is considered to have minimal effects). To maintain consistency in expectations, all participants were informed that they would be receiving psilocybin, but the specific dose was not disclosed. All participants received psychological support, consisting of therapeutic guidance and emotional care provided by two experienced clinicians before, during, and after psilocybin dosing sessions to ensure patient safety, integration of the experience, and management of any psychological distress. A Schedule 1 drug license from the UK Home Office was obtained, and the trial was approved by the Brent Research Ethics Committee, the U.K. Medicines and Healthcare Products Regulatory Agency, the Health Research Authority, the Imperial College London Joint Research Compliance and General Data Protection Regulation Offices, and the risk assessment and trial management review board at the trial site (the National Institute for Health Research Imperial Clinical Research Facility). Psilocybin was provided by COMPASS Pathways, and escitalopram and placebo were provided by the Pharmacy Manufacturing Unit at Guy’s and St. Thomas’s Hospital.

Participants had a preparatory session prior to dosing and two integration sessions following their dosing sessions. The first dosing session occurred the day after baseline, while the second dosing session occurred 3 weeks after the first dosing session. During dosing sessions, participants were administered either 25mg psilocybin (psilocybin group) or 1mg psilocybin (escitalopram group). Dosing sessions lasted approximately 6–8 hours and took place in a comfortable and aesthetically pleasing setting designed to feel non-clinical. The setting included a reclining bed, eyeshades, and a pre-set playlist of calming instrumental music. Participants were encouraged to adopt an open, accepting mindset. Study staff maintained a non-directive presence, offering support only as needed. At the end of the first dosing session, participants who had received 25mg of psilocybin on the first dosing day were given daily placebo capsules, while those who had received 1mg of psilocybin were given daily capsules of escitalopram (10mg following the first dosing session and 20mg following their second dosing session).

At baseline and post-treatment (3 weeks after their second dosing session), participants completed measures of mental health. The day after each dosing session, participants completed measures of their dosing session experience.

### Participants

Individuals between the ages of 18 and 80 years were recruited through trial networks, social media, and through other sources. Inclusion criteria were: 1) a score of at least 17 (indicating moderate-to-severe depression) on the 17-item Hamilton Depression Scale (HAM-D-17; Hamilton, 1960); and 2) confirmation of a diagnosis of major depressive disorder from the patient’s general physician. For exclusion criteria, see Supplementary Material. Patients were required to discontinue any psychiatric medications for at least 2 weeks and any psychotherapy for at least 3 weeks before starting trial medication.

### Measures

#### Demographics

At baseline, participants provided demographic information, including age, gender, race, education level, and employment status.

#### Acute Experience Measures

##### Experiences of Specific Positive Emotions

On the day of each dosing session, after the effects of the drug had worn off, specific positive emotions, mystical experience, and positive mood were measured using the same methodology as described in Study 1.

## Mental Health Outcomes

### Depression Severity

The primary clinical outcome in the parent trial was depression severity as measured using the Quick Inventory of Depressive Symptoms (QIDS-SR; Rush et al., 2003). The QIDS-SR is a 16-item self-report measure that assesses the presence and severity of the nine DSM-5 symptoms of major depression over the previous 7 days. The total score is calculated based on nine symptom domains, with the highest score used when multiple items assess the same domain (e.g., for sleep and appetite/weight disturbance). Items are rated on a scale from 0 to 3.

#### Suicidal Ideation

Suicidal ideation was assessed using the Suicidal Ideation Attributes Scale (SIDAS; Van Spijker et al., 2014), a five-item self-report measure evaluating the frequency, controllability, proximity to an attempt, distress level, and impact of suicidal thoughts on daily functioning. Responses are rated on a scale from 0 (e.g., “never”) to 10 (e.g., “always”), with one item reverse scored. The first item serves as a screening question (“In the past month, how often have you had thoughts about suicide?”), and individuals who select 0 (“never”) receive a total score of 0.

#### Trait Anxiety

Trait anxiety was assessed using the Trait Anxiety Scale of the State-Trait Anxiety Inventory (STAI-Trait; Spielberger, 1983). This 20-item self-report measure evaluates an individual’s tendency to experience anxiety in response to stressful situations. Responses are rated on a Likert scale from 1 (“not at all”) to 4 (“very much so”).

### Well-Being

#### Well-being was measured using the WEMWBS (see Methods of Study 1).

##### Statistical Analysis

A MANOVA examined the effect of psilocybin dose (25mg vs. 1mg) on maximum scores for positive emotional experiences. Statistical significance was set at *p*<.05 with false discovery rate FDR correction for multiple comparisons. Effect sizes are reported using partial eta squared *ηp*^*2*^. Exploratory analyses examined positive emotion scores from the first and second dosing sessions separately.

Pearson correlation analyses examined the relationships between maximum acute positive emotional experiences, mystical experience (MEQ total score), and positive mood (MEQ Positive Mood subscale) with residualized changes in mental health outcomes (i.e., depression severity, suicidal ideation, trait anxiety, and well-being) from baseline to post-treatment. We generated bias-corrected and accelerated (BCa) 95% confidence intervals (CI) using bootstrapping (with 5,000 resamples).

To investigate the mediating role of specific positive emotional experiences on the relationship between treatment condition (psilocybin vs. escitalopram) and improvements in mental health outcomes, we used the PROCESS Macro (Model 4; Hayes et al., 2022). Separate models were run to examine the effects of each mediator (i.e., self-compassion, compassion, gratitude, love, awe, ecstasy, and peace) with residualized change in each outcome variable. Multiple comparisons were corrected for using the Bonferroni method at the level of treatment outcomes (i.e., four analyses for each positive emotion and use of 98.75% confidence intervals). Bootstrapping (5000 samples) was used and indirect effects with confidence intervals that did not include zero were considered significant (Preacher & Hayes et al., 2008).

Exploratory analyses examined the unique effects of specific positive emotions above and beyond mystical experience and positive mood. In cases where a significant indirect effect was identified, the above analyses were repeated (without correction for multiple comparisons and using 95% confidence intervals) with either mystical experience or positive mood added separately as an additional mediator.

All statistical analyses were conducted using SPSS (Version 29.0.2.0). Figures were generated using R-studio (with the following libraries: ggplot2, scales, devtools, ggradar, & fmsb) and edited using GIMP photo-editing software.

## Study 2 (Randomized Controlled Trial): Results

### Descriptives

Participants’ mean age was 41.22 (*SD*=10.91). The majority of participants were male (*n*=39; 66.1%), White (*n*=52; 88.1%), employed (*n*=41; 69.5%), and had a university level of education (*n*=45; 76.3%). The mean duration of major depressive disorder was 18.66 years and the majority of individuals (*n*=32; 54%) had severe to very severe depression severity (as measured via the QIDS-SR) at baseline.

### Experiences of Specific Positive Emotions (25mg vs. 1mg Psilocybin)

**Figure 2:**
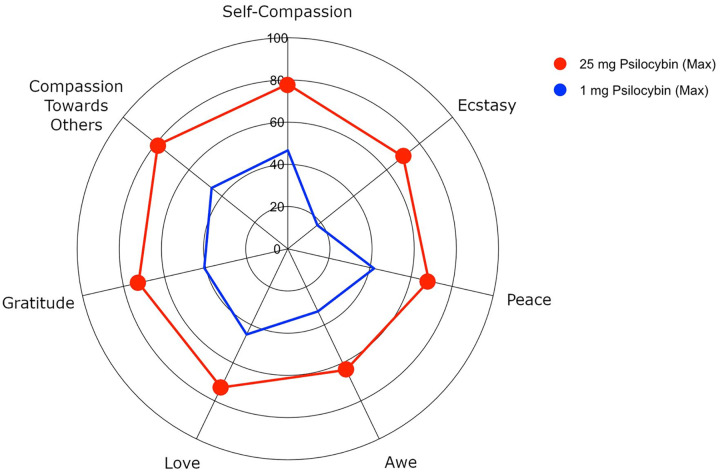
Spider plot demonstrating maximum ratings (across both dosing sessions) for each positive emotion (Study 2). The red lines indicate maximum 25mg dose ratings, and the blue lines indicate maximum 1mg dose ratings. Where ratings for 25mg differ significantly (*p*<.05) from ratings for 1mg, 25mg dose scores are marked with a filled in circle.

There was a significant effect of condition on maximum positive emotion scores, Pillai’s Trace=0.52, *F*(7,51)=7.88, *p*<.001, *ηp*^*2*^=.519. Relative to 1mg psilocybin, 25mg psilocybin was associated with significantly greater maximum scores for all positive emotions with medium (love and peace) to large (self-compassion, compassion toward others, gratitude, awe, and ecstasy) effect sizes. See Table 3 and Figure 2. For positive emotion experiences separated by dosing sessions, see Supplementary Materials (Table S2).

### Associations Between Positive Emotional Experiences and Treatment Outcomes

See Table 4 for results of the correlation analysis. Within the psilocybin condition, greater experiences of self-compassion, compassion toward others, awe, and peace were associated with reductions in depression severity and suicidal ideation. Awe was also associated with increases in well-being. Love was associated with improvement in depression severity, anxiety, and well-being. Effect sizes were large for the relationship between self-compassion with depression severity and suicidal ideation and medium for all other significant relationships.

### Positive Emotional Experiences as a Mediator of Treatment Outcomes

See Table 5 for results of the mediation analyses. Self-compassion was the only positive emotional experience that mediated the relationship between conditions (psilocybin vs. escitalopram) and improvements across all treatment outcomes. In models that included either mystical experience or positive mood, self-compassion remained the only significant mediator. Peace mediated the effects on depression severity, suicidal ideation, and well-being. Awe mediated the effects on suicidal ideation and well-being (including in models that included positive mood but not models that included mystical experience). Gratitude mediated the effects on suicidal ideation (including in models that included mystical experience or positive mood). Love mediated the effects on anxiety (even in models that included mystical experience and positive mood). Compassion toward others and ecstasy did not mediate the effects on any treatment outcomes. Neither mystical experience nor positive mood were significant mediators in any of the models where they were included alongside a specific positive emotion.

## Discussion

Little is known about the specific positive emotional experiences that psychedelics produce nor their role in facilitating mental health improvements. Across two studies, we found that relative to a low dose, a medium to high dose of a psychedelic is associated with a wide range of acute positive emotional experiences. Furthermore, we found that certain psychedelic induced positive emotional experiences are predictive of improvements in mental health and well-being. We found the strongest and most consistent evidence for the role of self-compassion (above and beyond mystical experience and positive mood), suggesting that specific positive emotional experiences may play an especially important role within psychedelic therapy.

The present studies are among the first to provide quantitative evidence for psychedelic use/psilocybin administration producing acute experiences across several specific positive emotional experiences. In the naturalistic study, we found that medium and high doses of psychedelics were associated with greater experiences across all positive emotional experiences relative to low doses. Preliminary results also suggest that the experience of specific positive emotions (e.g., love and gratitude) may be higher after psilocybin and ayahuasca administration compared to LSD. In the randomized controlled trial, 25mg psilocybin was associated with greater maximum levels of all positive emotional experiences across the two dosing sessions. While psychedelic research often focuses on the more non-ordinary elements of the psychedelic experience and their relation to long-term effects, we suggest that the effects of psychedelics on specific positive emotions is independently noteworthy and that these rich experiences may be intrinsically valuable (Oishi & Westgate, 2025).

With regards to their clinical relevance, several positive emotions mediated the effects of treatment (psilocybin therapy vs. escitalopram) on mental health outcomes. Self-compassion mediated all outcomes, while peace, awe, gratitude, and love selectively mediated specific therapeutic effects. The findings are line with interventional research suggesting that positive emotions play an important role in facilitating improved mental health (Craske et al., 2024) and that positive experiences may create upward emotional spirals that counteract maladaptive affective processes and widen attention, affect, and behaviors toward flourishing and living a meaningful life (Garland et al., 2010). These results are also in line with previous suggestions and qualitative research highlighting the importance of positive emotions within psychedelic therapy (e.g., Agin-Liebes, Nielson et al., 2024; Hendricks et al., 2018) Our findings add nuance and greater specificity to understanding how these positive emotions relate to treatment outcomes. While experience of peace did mediate several treatment outcomes in the clinical trial, it did not significantly correlate with improved well-being in the naturalistic sample. Additionally, while awe was associated with improvements in well-being following psychedelic use, as well as depression severity and suicidal ideation following psilocybin therapy, it did not mediate the effects of psilocybin therapy on depression severity or trait anxiety. The present study was the first psychedelic research to provide quantitative evidence for the relationship between love and compassion toward others and improved mental health, with love also mediating the psilocybin-induced reductions in trait anxiety. Interestingly, ecstasy was not correlated with well-being in the naturalistic study and did not correlate with or mediate any of the treatment outcomes in the clinical trial. In tandem with the findings that positive mood was not a key predictor of therapeutic improvement, these results suggest that it may not be the mere experience of positive mood or ecstasy/pleasure that drive the benefits of psychedelic therapy but the occurrence of transformative emotional experiences.

In the majority of models that included both a specific positive emotion and either mystical experience or positive mood, several positive emotions mediated the relationship (while mystical experience and positive mood did not). These findings highlight the contribution of specific positive emotions within psychedelic therapy. Accordingly, while ongoing debates regarding the importance of the acute psychedelic experience tend to center around non-ordinary elements of the psychedelic experience (Kush et al., 2025), the acute experience may also be important because it includes an amplification or enhancement of more ordinary experiences (that occur with less intensity within clinical samples). Ultimately, the present findings point to the importance of cross-talk between psychedelic research and other disciplines, including affective science and psychotherapeutic research (Aday et al., 2024).

“I had an encounter with a being, with a strong feeling that that was myself, telling me it’s alright, I don’t need to be sorry for all the things I’ve done. **I had an experience of tenderness towards myself**. During that experience, t**here was a feeling of true compassion I had never felt before**.”

• Psilocybin-assisted therapy for depression participant (Watts et al., 2017, p. 531)

Notably, self-compassion was the only positive emotion that mediated the relationship between treatment conditions (psilocybin therapy vs. escitalopram) and all mental health outcomes. These findings are in line with qualitative research (Agin-Liebes, Nielson et al., 2024) and expert consensus (Johansen et al., 2023), as well psychotherapeutic research that has emphasized the importance of self-compassion in driving therapeutic improvement (e.g., Nardone et al., 2024; Tani et al., 2025). When examined alongside mystical experience or positive mood, self-compassion was consistently a significant predictor and mediator of mental health outcomes while mystical experience and positive mood were not. This suggests that while the dissolution of one’s sense of self, a primary feature of the mystical experience, may be important, its importance may be dependent upon concurrent or subsequent experiences of altered *attitudes* or *emotional expression* towards one’s self. In other words, it may not be the breakdown or collapse of one’s self-model alone that matters, but the co-occurrence of self-compassion alongside this process or with the eventual reemergence of the sense of self. These results may help to explain the transdiagnostic potential of psychedelic therapy (Kočárová et al., 2021) and changes in self-attitudes (e.g., decreases in negative self-beliefs and increases in self-compassion) observed following psychedelic administration (e.g., Agin-Liebes et al., 2025; Khubsing et al., 2024; Zeifman et al., 2025). Namely, acute experiences of self-compassion may help facilitate a process of shifting deeply entrenched (see Carhart-Harris et al., 2023) self-attitudes away from being self-critical toward being more self-compassionate, and thereby toward improved mental health.

### Clinical Implications

The findings provide evidence for the acute psychedelic experience playing an important role in shaping treatment outcomes. In line with the context dependency (Carhart-Harris et al., 2018) and suggestibility-related (Carhart-Harris et al., 2015; Szigeti et al., 2024) effects of psychedelics, elements surrounding the psychedelic experience likely affect the tendency of specific emotional experiences to occur and their role in shaping treatment outcomes. For instance, experiences of peace, love, and self-compassion may be less likely to occur when individuals are administered a psychedelic in a cold and uncaring environment (e.g., see Smart et al., 1966). There may also be a potential role for integrating specific activities (e.g., time in nature to elicit experiences of awe; Ballew et al., 2018), therapeutic techniques (e.g., mindful self-compassion [Germer & Neff, 2019]), or psychotherapeutic approaches (e.g., compassion-focused therapy; Gilbert, 2010) within psychedelic therapy interventions. Based on the present findings, we suggest that increasing emphasis on the potential for these more ordinary emotional experiences may help to offset confusion and disappointment (e.g., among those who do not have a mystical experience); mainstream psychedelic therapy; and help maintain emphasis on the acute experience and surrounding psychological support. Focusing on positive emotional experiences throughout treatment may also help direct the therapeutic process toward making meaning of experiences that are more easily comprehended, experienced, and built upon in an individual’s daily life.

### Limitations and Future Directions

The present research should be considered in light of important methodological limitations. Not all specific positive emotions (e.g., feelings of power or lust) were assessed and positive emotions were assessed retrospectively at a single time point using a single-item self-report measure. Although measurement of positive emotions, including awe (e.g., Bai et al., 2017) and self-compassion (Rowe et al., 2016), with a single item is common, future research should measure specific positive emotions more extensively and should incorporate multi-item and multi-dimensional measures (e.g., the State-Trait Scales for Distinct Positive Emotions; Weidman & Tracy, 2020). Research should also use additional measurement approaches, such as micro-phenomenological analysis (Timmerman et al., 2023), facial expression, auditory, neurobiological, and physiological measurement. Observer-rated coding, experience sampling, and real time ratings of emotion may also enhance measurement sensitivity and allow for more nuanced measurement of blended emotions (e.g., anxiety mixed with feelings of peace; Lane et al., 2022), emotional sequences (e.g., grief followed by self-compassion; Pascual-Leone, 2018), and emotions accompanied by cognitive insight (Hayes et al., 2007). Study 1 was a naturalistic study, which may have been prone to imprecise quantification of psychedelic dose, selection biases, and expectancy effects. Although Study 2 was a randomized double-blind trial, it remains likely that a majority of participants were functionally unblinded (Wen et al., 2024). Additional clinical trials with more representative samples and diverse psychiatric presentations are also needed to establish the replicability and extension of the present findings (Zeifman & Maia, 2025).

In conclusion, the present findings provide evidence for classic psychedelics, and psilocybin specifically, producing acute increases in a wide range of positive emotional experiences. They provide evidence for the importance of the acute psychedelic experience in mediating the potential therapeutic benefits of psychedelic therapy and specifically support the importance of several positive emotional experiences, especially self-compassion. Accordingly, placing attention on the experience and integration of specific positive emotional experiences may be important for guiding the therapeutic process and optimizing the benefits of psychedelic therapy.

## Supplementary Material

Supplementary Files

This is a list of supplementary files associated with this preprint. Click to download.

- SupplementaryMaterials.docx

## Figures and Tables

**Table 1.** Relationship Between Psychedelic Dose and Positive Emotional Experiences (Study 1)

Variable	Low Dose Mean (*SD*)	Medium Dose Mean (*SD*)	High Dose Mean (*SD*)		Statistic	*p*	*p*_*fdr*_	*ηp* ^*2*^
Self-Compassion	41.72 (34.35)	60.35 (35.45)	64.95 (34.15)	Main Effect	6.80	.001	.001	.037
				Medium vs. low dose	2.87	.004	.006	0.54
				High vs. low dose	3.68	<.001	.001	0.67
				High vs. medium dose	1.18	.240	.280	0.13
Compassion toward others	34.69 (32.07)	64.17 (33.72)	67.67 (32.74)	Main Effect	15.16	<.001	<.001	.078
				Medium vs. low dose	4.76	<.001	<.001	0.89
				High vs. low dose	5.48	<.001	<.001	1.00
				High vs. medium dose	0.94	.347	.365	0.11
Gratitude	38.86 (33.56)	63.54 (34.45)	65.51 (33.24)	Main Effect	9.62	<.001	<.001	.051
				Medium vs. low dose	3.90	<.001	<.001	0.73
				High vs. low dose	4.34	<.001	<.001	0.79
				High vs. medium dose	0.52	.605	.605	0.06
Love	36.94 (33.09)	63.39 (35.56)	67.06 (33.15)	Main Effect	11.84	<.001	<.001	.062
				Medium vs. low dose	4.14	<.001	<.001	0.78
				High vs. low dose	4.86	<.001	<.001	0.88
				High vs. medium dose	0.96	.340	.365	0.11
Ecstasy	36.67 (30.80)	54.71 (30.04)	63.64 (27.90)	Main Effect	14.09	<.001	<.001	.073
				Medium vs. low dose	3.32	.001	.002	0.62
				High vs. low dose	5.11	<.001	<.001	0.93
				High vs. medium dose	2.73	.007	.009	0.31
Awe	29.78 (32.96)	58.17 (35.17)	68.47 (32.60)	Main Effect	20.58	<.001	<.001	.104
				Medium vs. low dose	4.50	<.001	<.001	0.84
				High vs. low dose	6.32	<.001	<.001	1.15
				High vs. medium dose	2.72	.007	.009	0.31
Peace	37.19 (32.96)	59.82 (33.98)	67.06 (32.75)	Main Effect	12.41	<.001	<.001	.065
				Medium vs. low dose	3.63	<.001	.001	0.68
				High vs. low dose	4.94	<.001	<.001	0.90
				High vs. medium dose	1.93	.054	.067	0.22

**Table 2.** Associations Between Positive Emotional Experiences and Residualized Change in Well-Being (Study 2)

Acute Experience	Pearson Correlation		Linear Regression			
				Positive Emotions Only	Positive Emotions and Mystical Experience	Positive Emotions and Positive Mood lncluded
1. Self-Compassion	*r*	**.36**	*β*	**0.30**	**0.31**	**0.31**
	95% BCa CI	**0.21, 0.50**	95% BCa CI	**0.01, 0.11**	**0.01, 0.11**	**0.01, 0.11**
2. Compassion Toward Others	*r*	**.35**	*β*	0.26	0.27	0.26
	95% BCa CI	**0.19, 0.49**	95% BCa CI	−0.00, 0.09	−0.00, 0.10	−0.01, 0.10
3. Gratitude	*r*	**.24**	*β*	−0.05	−0.02	.−0.08
	95% BCa CI	**0.07, 0.39**	95% BCa CI	−0.05, 0.03	−0.05, 0.04	−0.06, 0.03
4. Love	*r*	**.23**	*β*	0.11	−0.10	.−0.11
	95% BCa CI	**0.08, 0.05**	95% BCa CI	−0.78, 0.00	−0.07, −0.04	−0.07, 0.03
5. Ecstasy	*r*	.09	*β*	−0.06	−0.04	−0.14
	95% BCa CI	−0.08, 0.25	95% BCa CI	−0.06, 0.03	−0.05, 0.04	−0.10, 0.04
6. Awe	*r*	**.18**	*β*	0.07	0.07	0.04
	95% BCa CI	**0.02, 0.34**	95% BCa CI	−0.03, 0.05	−0.02, 0.05	−0.03, 0.05
7. Peace	*r*	.15	*β*	−0.08	−0.06	−0.10
	95% BCa CI	−0.03, 0.32	95% BCa CI	−0.05, 0.03	−0.05, 0.03	−0.06, 0.03
8. Mystical Experience (MEQ total)	*r*	.15	*β*	--	−0.09	--
	95% BCa CI	−0.05, 0.33	95% BCa CI		−0.10, 0.06	
9. Positive Mood (MEQ subscale)	*r*	.18	*β*	--	--	−0.15
	95% BCa CI	−0.03, 0.34	95% BCa CI			−0.08, 0.17

Note. BCa CI = Bias-corrected and accelerated Confidence Intervals. Bolded statistics denote statistical significance based on 95% BCa CI.

**Table 3.** Maximum Scores for Experiences of Specific Positive Emotions (25mg vs. 1mg Psilocybin; Study 2)

Variable	25mg Psilocybin Mean (SD)	1mg Psilocybin Mean (SD)	*F*	*p*	*p*_*fdr*_	*ηp* ^*2*^
Self-Compassion	64.30 (40.08)	29.97 (35.46)	10.08	.002	.004	.150
Compassion toward others	72.03 (36.67)	25.52 (34.59)	11.91	.001	.002	.173
Gratitude	58.43 (41.54)	27.10 (30.14)	12.02	.001	.002	.174
Love	58.60 (40.34)	28.83 (34.90)	8.77	.004	.005	.133
Ecstasy	59.33 (36.57)	12.41 (18.83)	46.62	<.001	<.001	.450
Awe	58.67 (40.80)	17.28 (26.64)	9.86	.003	.004	.147
Peace	68.67 (36.30)	41.97 (34.51)	8.38	.005	.005	.128

**Table 4.** Associations Between Positive Emotional Experiences (Maximum Scores Across Dosing Sessions) and Treatment Outcomes (Study 2)

	Psilocybin Condition					Escitalopram Condition			
		ΔDepression Severity	ΔSuicidal Ideation	ΔTrait Anxiety	ΔWell-Being	ΔDepression Severity	ΔSuicidal Ideation	ΔTrait Anxiety	ΔWell-Being
1. Self-Compassion	*r*	**−.50**	**−.52**	−.39	.37	**−.36**	**−.39**	**−.42**	**.53**
	95% BCa CI	**−0.80, −0.06**	**−0.80, −0.14**	−0.70, 0.11	−0.04, 0.67	**−0.62, −0.04**	**−0.61, −0.11**	**−0.66, −0.12**	**0.24, 0.75**
2. Compassion Toward Others	*r*	**−.48**	**−.47**	−.41	.38	−.10	−.26	−.21	.17
	95% BCa CI	**−0.77, −0.08**	**−0.79, −0.08**	−0.71, 0.04	−0.07, 0.70	−0.44, 0.29	−0.53, 0.15	−0.53, 0.12	−0.24, 0.62
3. Gratitude	*r*	−.33	−.40	−.25	.24	−.26	**−.39**	**−.31**	**.30**
	95% BCa CI	−0.69, 0.13	−0.76, 0.03	−0.62, 0.24	−0.13, 0.54	−0.58, 0.09	**−0.60, −0.11**	**−0.58, −0.06**	**.012, 0.60**
4. Love	*r*	**−.46**	−.44	**−.47**	**.42**	−.25	−.34	**−.37**	.34
	95% BCa CI	**−0.78, −0.31**	−0.78, 0.00	**−0.73, −0.03**	**0.37, 0.69**	−0.61, 0.14	−0.61, 0.03	**−0.66, −0.01**	−0.11, 0.75
5. Ecstasy	*r*	−.31	−.22	−.33	.27	**−.40**	−.17	−.37	.17
	95% BCa CI	−0.66, 0.14	−0.57, 0.10	−0.63, 0.07	−0.08, 0.59	**−0.65, −0.06**	−0.46, 0.24	−0.63, 0.03	−0.18, 0.47
6. Awe	*r*	**−.42**	**−.48**	−.32	**.36**	−.23	**−.31**	−.30	.37
	95% BCa CI	**−0.73, −0.07**	**−0.73, 0.19**	−0.63, 0.06	**0.23, 0.65**	−0.55, 0.14	**−0.55, −0.003**	−0.60, 0.09	−0.01, 0.70
7. Peace	*r*	**−.40**	**−.44**	−.30	.24	**−.42**	**−.42**	**−.40**	**.52**
	95% BCa CI	**−0.68, −0.07**	**−0.68, −0.20**	−0.62, 0.09	−0.11, 0.56	**−0.69, −0.08**	**−0.60, −0.18**	**−0.64, −0.09**	**0.24** **0.75**
8. Mystical Experience (MEQ total)	*r*	−.42	**−.56**	−.33	**.40**	−.34	−.18	−.26	.22
	95% BCa CI	−0.72, 0.00	**−0.78, −0.28**	−0.61, 0.02	**0.09, 0.65**	−0.60, 0.00	−0.48, 0.26	−0.53, 0.09	−0.15, 0.56
9. Positive Mood (MEQ subscale)	*r*	−.30	**−.34**	−.25	.27	**−.40**	−.24	−.37	.27
	95% BCa CI	−0.67, 0.07	**−0.65, −0.07**	−0.57, 0.08	−0.3, 0.59	**−0.66, −0.06**	−0.53, 0.18	−0.62, 0.01	−0.10, 0.59

Note. Δ=Residualized change. BCa CI = Bias-corrected and accelerated Confidence Intervals. Bolded statistics denote statistical significance based on 95% BCa CI.

**Table 5.** Indirect Effects for Specific Positive Emotions as Mediators of Treatment Outcomes (Study 2)

Mediator(s)	Treatment Outcome											
	Depression Severity			Suicidal Ideation			Trait Anxiety			Well-Being		
	Indirect Effect	*SE*	CI	Indirect Effect	*SE*	CI	Indirect Effect	*SE*	CI	*indirect Effect*	*SE*	CI
Self-Compassion	**−1.92**	**0.84**	**−4.59, −0.22**	**−1.84**	**0.89**	**−4.72, −0.22**	**−3.65**	**1.67**	**−8.94, −0.32**	**3.66**	**1.55**	**0.62, 8.28**
Self-Compassion	**1.46**	**0.81**	**−3.27, −0.12**	**−1.56**	**0.82**	**−3.48, −0.28**	**−3.01**	**1.67**	**−6.74, −0.17**	**3.04**	**1.54**	**0.52, 6.47**
Mystical Experience	−2.48	1.38	−4.94, 0.49	−1.50	1.26	−3.94, 1.03	−3.44	2.83	−9.08, 2.20	3.34	2.43	−1.76. 7.92
Self-Compassion	**−1.55**	**0.82**	**−3.28, −0.83**	**−1.65**	**0.82**	**−3.52, −0.35**	**−3.06**	**1.64**	**−6.77, −0.28**	**3.26**	**1.65**	**0.68, 7.01**
Positive Mood	−2.19	1.65	−5.50, 0.82	−1.14	1.41	−4.02, 1.62	−3.58	3.30	−10.73, 1.93	2.44	2.61	−3.25, 7.34
Compassion Toward Others	−1.37	0.81	−3.87, 0.55	−1.49	0.89	−4.33, 0.20	−3.11	1.70	−8.16, 0.52	2.52	1.49	−0.73, 7.10
Gratitude	−1.48	0.85	−4.05 0.24	**−1.77**	**0.92**	**−4.91, −0.19**	−2.79	1.76	−8.10, 0.90	2.45	1.38	−0.46, 6.60
Gratitude	--	--	--	**−1.40**	**0.84**	**−3.42, −0.17**	--	--	--	--	--	--
Mystical Experience				−1.61	1.31	−4.22, 1.09						
Gratitude	--	--	--	**−1.55**	**0.84**	**−3.53, −.29**	--	--	--	--	--	--
Positive Mood				−0.81	1.56	−4.10, 2.22						
Love	−1.52	0.77	−3.92, 0.09	−1.48	0.81	−4.08, 0.02	**−3.63**	**1.64**	**−8.84, −0.35**	2.99	1.44	−0.07, 7.34
Love	--	--	--	--	--	--	**−3.08**	**1.68**	**−6.34, −0.27**	--	--	--
Mystical Experience							−2.79	2.87	−8.41, 3.05			
Love	--	--	--	--	--	--	**−3.11**	**1.63**	**−6.67, −0.32**	--	--	--
Positive Mood							−2.87	3.35	−10.32, 2.56			
Ecstasy	−3.31	1.34	−6.50, 0.30	−1.55	1.15	−5.10, 0.92	−6.80	2.95	−15.01, 0.39	4.38	2.24	−1.50, 10.40
Awe	−1.52	0.83	−4.21, 0.01	**−1.50**	**0.78**	**−4.21, −0.13**	−2.87	1.79	−8.68, 0.31	**3.07**	**1.50**	**0.30, 7.70**
Awe	--	--	--	−1.12	0.76	−2.91, 0.03	--	--	--	2.23	1.62	−0.28, 6.03
Mystical Experience				−1.31	1.58	−4.36, 2.01				2.92	2.83	−3.29, 7.90
Awe	--	--	--	**−1.32**	**0.81**	**−3.29, −0.13**	--	--	--	**2.70**	**1.81**	**0.10, 7.04**
Positive Mood				−0.65	1.83	−4.57, 2.74				1.35	3.20	−5.62, 6.99
Peace	**−1.68**	**0.85**	**−4.39, −0.17**	**−1.60**	**0.86**	**−4.53, −0.11**	−2.89	1.72	−8.66, 0.07	**2.77**	**1.45**	**0.16, 7.27**
Peace	−1.14	0.87	−3.07, 0.30	**−1.37**	**0.85**	**−3.44, −0.19**	--	--	--	1.96	1.60	−0.52, 5.59
Mystical Experience	−2.15	1.70	−5.29, 1.48	−0.92	1.60	−3.69. 2.57				3.23	3.25	−4.04. 9.25
Peace	**−1.29**	**0.90**	**−3.40, −0.01**	**−1.58**	**0.90**	**−3.79, −0.28**	--	--	--	**2.39**	**1.74**	**0.01, 6.63**
Positive Mood	−1.50	2.02	−5.41, 2.47	−0.09	1.79	−3.55, 3.55				1.47	3.42	−5.88, 7.80

Note. Indirect effect = *a* path × *b* path, where the *a* path is the effect of condition (psilocybin vs. escitalopram) on the acute experience variable and the *b* path is the effect of the acute experience variable on the treatment outcome. SE = Standard error. CI = Confidence interval. In models that only include a specific positive emotion, confidence intervals are 98.75%. When models include mystical experience or positive mood, confidence intervals are 95%. Bolded statistics denote statistical significance.
